# Comparison of antibacterial and antioxidant potentials of pure and nanoemulsified *Nepeta pogonosperma* essential oil

**DOI:** 10.1002/fsn3.3210

**Published:** 2022-12-28

**Authors:** Kimia Sharifi, Akram Sharifi

**Affiliations:** ^1^ Department of Food Science and Technology Qazvin Branch Islamic Azad University Qazvin Iran

**Keywords:** antibacterial properties, emulsification, GC/MS, IC_50_, *Nepeta pogonosperma*

## Abstract

The current study aimed to investigate the antiradical and antibacterial potential of pure and its nanoemulsified (NNE) *Nepeta pogonosperma* essential oil (PNE). Antimicrobial activity of the essential oil against two Gram‐positive (*E. faecalis* and *B. cereus*) and two Gram‐negative (*M. catarrhalis* and *K. pneumonia*) food‐related pathogens during 60‐day storage was investigated based on disc diffusion, minimum inhibition concentration (MIC), and minimum bactericidal concentration (MBC). The chemical compounds of Nepeta *essential oil* were estimated by GC/MS. The physical properties of the nanoemulsion including polydispersity index (PDI), mean particle diameter, and viscosity were also determined. 4aα,7α,7aβ‐Nepetalactone (46.31%), 1,8‐cineole (23.13%), and (Z)‐α‐bisabolene (4.01%) were the main compounds of this essential oil. The Nepeta nanoemulsion had a mean droplet diameter of 254.07 nm, PDI of 0.281, and viscosity of 0.887 cP. NNE had stability for up to 60 days. The PNE showed a higher IC_50_ value than NNE (*p* < .05). During storage, the antiradical performance of both PNE and NNE was decreased (*p* < .05). However, emulsification was successful to control this decreasing trend. *E. faecalis* was the most susceptible bacteria to PNE and NNE, while the lowest inhibition zone was obtained for *K. pneumoniae*. At the first time, the antibacterial effect of PNE was more than NNE. However, over time nanoemulsion became more successful in maintaining its antibacterial effect. Overall, the incorporation of *Nepeta pogonosperma* essential oil into a nanoemulsion system can be a promising system to maintain the bioactivity of the essential oil for a longer time.

## Graphical abstract

- 4aa,7a,7aß‐Nepetalactone (46.31%) was the main compounds of Nepeta essential oil.
- Nanoemulsion of Nepeta essential oil was prepared by ultrasonication.
- The nanoemulsion maintains its bioactivity over time.
- The antibacterial activity of the Nepeta nanoemulsion was better than the essential oil.

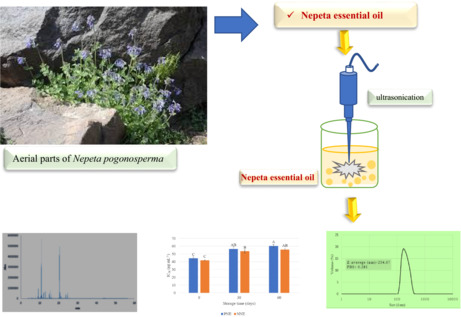

## 1 INTRODUCTION

Plant‐based bioactive compounds have attracted a great attention in food and nutraceutical industry due to their antimicrobial and antioxidant potentials (Shahidi et al., 2020; Sharifi et al., 2019; Tongnuanchan & Benjakul, 2014). Most of plant‐based essential oils are generally recognized as safe (GRAS) substances by the Food and Drug Administration and are approved as flavorings in the European Union (Sharma et al., 2019; Surianarayanan & Bhaskar, 2020). Essential oils are suitable additives due to the presence of valuable bioactive compounds with antimicrobial and antioxidant properties (Hashemi et al., 2013; Krishnaiah et al., 2011; Purkait et al., 2018).

The genus *Nepeta* L. (catmint) belongs to the *Lamiaceae* family and *Nepetoideae* subfamily with around 400 species (Asgarpanah et al., 2014). The majority of species of *Nepeta* L. are native to temperate Europe, North Africa, Asia, and North America (KiliÇ et al., 2007). One of the greatest Nepeta species diversity found in Iran by about 75 species (Jamzad, 2006; Javidnia et al., 2011) among them are *N. pungens*, *N. binaludensis*, *N. isphanica*, *N. pogonosperma* and *N. bracteata* (the most frequent Iranian folk medicine) (Hadi et al., 2017; Layeghhaghighi et al., 2017). *Nepeta pogonosperma* is a new species identified in 1984 (Jamzad & Assadi, 1984; Valimehr et al., 2014). The previous studies reported the chemical composition, antinociceptive, anti‐inflammatory, and antioxidant potential of *N. pogonosperma* (Afshar Mehrabi et al., 2021; Ali et al., 2012; Khalighi‐Sigaroodi et al., 2013; Sefidkon & Akbari‐Nia, 2003; Valimehr et al., 2015). The bioactivity of *Nepeta* species may be related to the terpenoid compounds (such as nepetalactones, sesquiterpenes, monoterpenes, and cyclopentanoid iridoid derivatives), phenolics, and flavonoids (Akdeniz et al., 2020; Formisano et al., 2011; Süntar et al., 2018). Essential oils show poor solubility in water, high volatility, and strong flavor as well as sensitivity to environmental stresses (e.g., pH, light, high temperature, oxygen, etc.) limiting their use in the food industry (Khoshnoudi‐Nia, Forghani, et al., 2020; Prakash et al., 2018). Encapsulation (entrapping one material within a vehicle in micro‐ or nanometric size) is a suitable technique to offer new formulations with higher water solubility and protect bioactive compounds against inactivation by harsh environmental conditions (Khoshnoudi‐Nia, Sharif, et al., 2020; Sharifi et al., 2015).

Emulsion‐based technology is the most attractive approach for the delivery of essential oils due to its easy and inexpensive preparation. In this method, hydrophobic material (e.g., essential oil) is encapsulated within colloidal‐based delivery systems by various techniques such as ultrasonication, microfludization, high pressure, and homogenization stabilized by surfactant molecules (McClements, 2011; Nishad et al., 2021; Yazgan, 2020). Among these techniques, the ultrasonication method as a green technology can obtain nanoemulsions with lower polydispersity index (PDI), higher stability, and smaller droplet size, using less amount of surfactant and energy consumption compared to microfludization and high‐pressure homogenization. Ultrasonic power produces oil droplets by generating intensive disruptive forces and breaking up the water and oil phases (Nirmal et al., 2018; Pongsumpun et al., 2020). Therefore, the ultrasonication method was used in the current study to prepare emulsion of Nepeta essential oil.

Nanoemulsion is an oil‐in‐water (o/w) or water‐in‐oil (w/o) emulsion with a mean droplet size of 20–200 nm. Nanoemulsions exhibit optical transparency, controlled releasing, suitable solubility, high physical stability, and bioactivity even at lower concentrations (Flores et al., 2011; Mahdi & Maraie, 2019; Rao & McClements, 2013). Several studies have compared the bioactivity of various pure essential oils (e.g., Verbenaceae, lemon myrtle and anise myrtle, grapefruit peel, lemongrass, and basil) and their nanoemulsions, and investigated the promising potential of nanoemulsions as an effective treatment for improving bioactivity of essential oils (Balasubramani et al., 2017; Kumar & Kumar, 2018; Nirmal et al., 2018; Prakash et al., 2018; Seibert et al., 2019; Sundararajan et al., 2018; Yazgan, 2020). However, the antibacterial and antioxidant potential of *N. pogonosperma* and essential oil nanoemulsion have not been studied. To the best of our knowledge, this is the first study addressing the antibacterial and antioxidant potential of *Nepeta pogonosperma* and nanoemulsions. Therefore, the current study aimed to investigate the antiradical potential of pure *Nepeta pogonosperma* and essential oil and its nanoemulsion. The antimicrobial activity of essential oils against two Gram‐positive (*E. faecalis* and *B. cereus*) and two Gram‐negative (*M. catarrhalis* and *K. pneumonia*) food‐related pathogens during 60 days of storage was studied to investigate the benefits of nanoemulsion compared to the pure form. Moreover, the chemical compounds of *Nepeta pogonosperma* were estimated by GC/MS. The properties of nanoemulsion such as polydispersity index (PDI), mean particle diameter, viscosity, and stability were also determined.

## 2 MATERIALS AND METHODS

### 2.1 Material

The aerial parts of *N. pogonosperma* were collected from the Alamut region (Qazvin province, Iran) in July 2020. A voucher specimen (MPIH‐527) has been deposited at the central herbarium of Tehran, Iran. The aerial parts of Nepeta were dried at shade (23 ± 3°C). Then, the air‐dried plant was milled (model 320P; Pars‐Khazar) and kept in a dark bottle. All chemicals in analytical grades were bought from Merck (Darmstadt, Germany) and Dr. Mojalali (Tehran, Iran). The bacterial strain cultures were purchased from the *Iranian Research Organization for Science and Technology* (IROST).

### 2.2 *Nepeta pogonosperma* essential oil extraction

The pure Nepeta essential oil (PNE) was extracted from the air‐dried plant (100 g) based on the hydrodistillation technique for 4 h using a Clevenger apparatus (Azmiran). The oil was dried with anhydrous Na_2_SO_4_ and kept in a sealed dark glass bottle at 4 ± 1°C until analysis (Sefidkon & Akbari‐Nia, 2003).

### 2.3 Nanoemulsion preparation

Oil‐in‐water nanoemulsion of Nepeta essential oil (NNE) was prepared from a mixture of *Nepeta pogonosperma* (10% w/w), Tween 80 (1% w/w), Span 80 (1% w/w), and water (88% w/w). Essential oil, Tween 80, and Spin 80 were GRAS (generally recognized as safe). An ultrasonic homogenizer (Topsonic, 400 W, 20 kHz, Iran; 15 min at 72 amplitudes) was applied to prepare the nanoemulsion. During this homogenization, the emulsion temperature was maintained at 15°C using ice around the beaker (Balasubramani et al., 2017).

### 2.4 Chemical composition of Nepeta essential oil

The volatile compound of essential oil was analyzed by gas chromatography–mass spectrometry (GC/MS) system Agilent 6890N/5973 inert (Agilent Technologies, USA) fitted with a DB‐1‐fused silica capillary column (60 m × 0.25 mm and 0.25 μm thickness). The injected volume was 1 μl and the split ratio was 1/50. The oven's initial temperature was increased (ramp rate: 5°C min^−1^) from 60 to 250°C and for 10 min held at the final temperature. The injector temperature was 220°C. Diluted Nepeta essential oil (1 μl) in hexane was injected into the column by using a split‐less injection technique. Mass spectra (MS) were scanned between 45 and 465 amu. Electron ionization was set at 70 eV and 150 μA. The ion source temperature was 200°C. The interface line temperature was 250°C. Individual components of the essential oil were identified by comparison of their mass spectra with those in WILEY‐MS libraries or reported in the literature. Quantitative data of the Nepeta volatile compounds obtained from GC‐FID areas percentages (Salehi et al., 2007).

### 2.5 Physical properties of nanoemulsion

The polydispersity index (PDI) and mean particle size of the emulsified droplets were measured by a dynamic light scattering (DLS) (Nanophox Sympatec GmbH, Germany) system based on a laser diffraction particle size analyzer at 25°C. Each sample was diluted with deionized water at a 1:20 ratio to avoid interparticulate interaction and multiple scatterings (Kumar & Kumar, 2018). The viscosity of nanoemulsion was measured by a rheometer (Stable Micro System, TA.XT2i England). The stability of nanoemulsion based on Nepeta essential oil was visually monitored over 60 days by considering color, phase separation, and creaming formation (Motta Felício et al., 2021).

### 2.6 Free radical scavenging capacity

The scavenging activity of Nepeta essential oil and its nanoemulsion were estimated on the 2,2‐diphenyl‐1‐picrylhydrazil (DPPH) radical. First, the essential oil or its nanoemulsion (0.1 ml) at various concentrations was mixed with 5‐ml methanolic solution of DPPH (4.5 ml of methanol and 0.5 ml of DPPH solution). After 1 h incubation at room temperature, the absorbance of the solutions was read at 517 nm (Shimadzu 2501UV spectrophotometer). The percentage of radical scavenging capacity (RSC) was calculated using the following equation:

$$\mathrm{RSC}\%=\left(A_{\text{blank}}-A_{\text{sample}}/A_{\text{blank}}\right)\times 100,$$

where *A*_blank_ and *A*_sample_ are control (methanolic solution of DPPH) and extract absorbance at 517 nm, respectively.

The IC_50_ value (50% inhibitory concentration) was evaluated using linear regression analysis from the obtained RSC values (Koleva et al., 2002).

### 2.7 Assignment of antibacterial activity

#### 2.7.1 Bacterial culture

The food‐related pathogens were used to evaluate the antimicrobial effect of the pure and emulsified Nepeta essential oil as follow: *Enterococcus faecalis* ATCC29212, *Klebsiella pneumoniae* ATCC700603, *Bacillus cereus* PTCC1247, and *Moraxella catarrhalis* ATCC700603.

#### 2.7.2 Bacterial inhibition assay

The bacterial inhibition effect of the Nepeta essential oil and its nanoemulsion was evaluated by agar disc diffusion assay (Murray et al., 1982) with some modifications. Bacterial suspension (1 ml; 10^8^ CFU ml^−1^) was spread on nutrient agar plates, and then, paper disc (diameter: 6 mm) was impregnated with 50 μl of Nepeta essential oil or its nanoemulsion. The discs were placed over plates of Muller Hinton agar (MHA, Difco) seeded with each bacterium. Turbidity was set to 0.5 Mc Farland standard. The plates were incubated at 37 ± 1°C for 24 h. The antibacterial effect was evaluated by measuring the zones of inhibition (mm) around each of the discs (Domig et al., 2007).

#### 2.7.3 Minimum inhibition/bactericidal concentration (MIC/MBC)

The MIC and MBC values of Nepeta essential oil and its nanoemulsion against food‐related pathogens were measured. Briefly, 1 ml of pure or emulsified essential oil (stock solution: 50 mg ml^−1^) was added to the first tube in each series and diluted with sterile Muller–Hinton Broth (MHB, Merck, Germany). Then, 1 ml of each bacterial suspension (10^6^ CFU ml^−1^) was added to each tube. The final concentrations of the sample were 50, 25, 12.5, 6.25, 3.12, 1.56, 0.78, and 0.19 mg ml^−1^. All samples were incubated at 35°C for 24 h. The tubes were investigated for turbidity of the medium as an indicator of microbial growth. The MIC values were defined as the lowest Nepeta essential oil concentration inhibiting visible growth of the tested microorganism. MBC was evaluated by subculturing the contents of tubes of MIC into Mueller‐Hinton Agar (MHA), which showed no growth (CLSI, 2008).

### 2.8 Statistical analysis

All analyses were performed in three replicates and the results were reported as mean values and standard division (mean ± SD). ANOVA/general linear model (GLM) was used for statistical analyses. The significant differences between the means were tested by the LSD test (*p* < .05). Statistical calculations were accomplished in Minitab software (version 17; Minitab Inc.).

## 3 RESULTS AND DISCUSSIONS

### 3.1 Chemical composition of Nepeta essential oil

**FIGURE 1:**
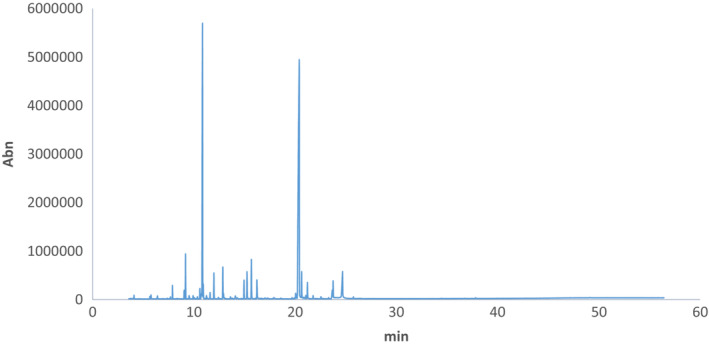
Chromatogram of *Nepeta pogonosperma* essential oil

The essential oil of *N. pogonosperma* was light‐yellow oil with a strong pleasant odor. GC/MS analysis determined the chemical compositions of *N. pogonosperma* essential oil (Table 1, Figure 1). Overall, 35 compounds were identified (98.45% of the total oil). Oxygenated monoterpenes above 80% of essential oil.

**TABLE 1:** Chemical composition of *Nepeta pogonosperma* essential oils

	Composition	Rt* (min)	%		Composition	Rt (min)	%
1	Methyl isovalerate	4.09	0.23	19	α‐Terpinolene	12.42	0.14
2	Ethyl 2‐methylbutyrate	5.66	0.12	20	Linalool	12.86	2.31
3	Ethyl isovalerate	5.77	0.21	21	γ‐Terpinen	13.61	0.20
4	2‐Methybutyl acetate	6.41	0.24	22	cis‐Sabinol	14.09	0.31
5	α‐Thujene	7.68	0.12	23	α‐Terpinol	14.95	1.23
6	α‐Pinene	7.88	0.73	24	Terpinen‐4‐ol	15.25	1.78
7	α‐Sabinene	9.03	0.45	25	α‐Terpineol	15.68	2.78
8	β‐Pinene	9.18	2.65	26	Isocreosol	16.23	1.59
9	β‐Phellandrene	9.53	0.30	27	4aα, 7α, 7aβ‐Nepetalactone	20.37	46.31
10	Hexyl valerate	9.92	0.17	28	cis‐geraniol	20.64	1.96
11	α‐Terpinen	10.35	0.16	29	1‐Methyl‐1‐(2‐methylprop‐2‐enyl) cyclopentane	21.06	0.24
12	o‐Cymol	10.59	0.70	30	4aβ, 7α, 7aβ‐Nepetalactone	21.22	1.20
13	Limonene	10.75	0.45	31	α‐Dihydronepetalactone	21.78	0.23
14	1,8‐Cineole^om^	10.8	23.13	32	β‐Farnesene	22.55	0.12
15	(E)β‐Ocimene	10.93	0.83	33	1‐Hexyl‐1‐cyclohexene	23.75	1.21
16	(Z)β‐Ocimene	11.25	0.16	34	(Z)‐α‐Bisabolene;	24.67	4.01
17	γ‐Terpinen	11.60	0.42	35	Dimethylsiloxane cyclic trimer	25.76	0.12
18	cis‐Sabinen hydrate (cis‐4‐Thujanol)	11.98	1.64		Total	98.45	

Abbreviation: Rt, retention time.

4aα,7α,7aβ‐Nepetalactone (oxygenated monoterpenes: 46.31%), 1,8‐cineole (oxygenated monoterpenes: 23.13%), (Z)‐α‐bisabolene (sesquiterpene hydrocarbons: 4.01%), α‐terpineol (oxygenated monoterpenes: 2.78), and β‐pinene (monoterpene hydrocarbons: 2.65) were the main compounds of this essential oil. Previous authors showed the antioxidant and antimicrobial potentials of these compounds (Mahajan et al., 2019; Paul & Bhattacharjee, 2018; Salehi et al., 2012; Sonboli et al., 2004). According to Sefidkon and Akbari‐Nia (2003), the main essential oil composition of *N. pogonosperma* are 4aα,7α,7aβ‐nepetalactone (57.6%) and 1,8‐cineol (26.4%) (Sefidkon & Akbari‐Nia, 2003). However, Ali et al. (2012) found 1,8‐cineole (31.2%), and 4aα,7α,7aα‐nepetalactone (14.5%), (E)‐α‐bisabolene (5.4%), α‐terpineol (5.4%), terpinen‐4‐ol (4.8%), linalool (4.5%), and β‐pinene (3.5%) as the major compounds. Moreover, Talebi et al. (2020) considered 1,8‐cineol (53.9%), 4aα,7α,7aα‐nepetalactone (6.2%), *Z*‐α‐bisabolene (5%), linalool (4.1%), and rpinen‐4‐ol (3.8%) as the predominant components of this species. It was reported that some environmental and geographical factors such as soil properties, ozone concentration, temperature, wind exposure, relative humidity, photoperiod, light intensity, and partial CO_2_ pressure as well as the genetic structure could significantly affect the secondary metabolites synthesis, and consequently, the chemical composition and yield of Lamiaceae essential oil (Kofidis & Bosabalidis, 2008; Layeghhaghighi et al., 2017; Talebi et al., 2019). For example, Layeghhaghighi et al. (2017) showed the significant effect of altitude on the quality and quantity of *Nepeta pogonesperma* essential oil collected from the Alamut region. The maximum percentage of 1,8‐cineole, ρ‐cymene, and nepetalactone was observed in 2400, 2600, and 2800 m, respectively.

### 3.2 Physical properties of *Nepeta pogonosperma* nanoemulsion

**FIGURE 2:**
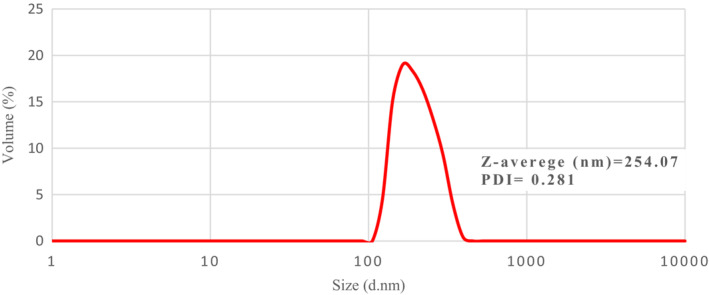
The particle size distribution of nanoemulsion based on *Nepeta pogonosperma* essential oil. PDI, polydispersity index.

The mean droplet size (Z‐average) and PDI of *Nepeta pogonosperma* nanoemulsion are presented in Figure 2. The Nepeta nanoemulsions had a relatively narrow size distribution with a single peak at around 250 nm. The particle size distribution of the droplets can be reported by the particle diameter. A small PDI value (PDI < 0.3) shows a narrow particle size distribution of the essential oil droplets in an aqueous system (Badran & Elsayed, 2013; Nguyen et al., 2018). PDI value of the *Nepeta pogonosperma* nanoemulsion was found as 0.281, indicating a suitable quality and homogeneity of the nanoemulsion. This result was comparable with several previous findings. For example, Lee et al. (2019) reported that the oregano nanoemulsions had a mean droplet diameter of 221 nm and PDI of 0.251 (Lee et al., 2019), and Yazgan et al. (2019) found a smaller value (PDI = 0.114) for the nanoemulsified lemon oil (Yazgan et al., 2019). The PDI values can be varied by different nanoemulsion formulations (especially type and concentration of emulsifier) and preparation conditions (Donsì et al., 2012; Kumar & Kumar, 2018).

In the current study, the mean particle diameter of the nanoemulsified essential oil was 254.07 nm. Moghimi et al. (2017) also reported relatively similar droplet sizes for the nanoemulsion of sage oil (10% w/v) prepared based on nonionic surfactants (Span 80: Tween 80 ratio of 1:1, and 2% w/v) using ultrasonication (Moghimi et al., 2017). However, the mean droplet size of emulsified lemon essential oil formulated with Tween 80 was 187.5 nm (Yazgan et al., 2019). Lee et al. (2019) pointed to the role of high‐energy shock waves of ultrasound to corroborate the reduction in the size of bigger droplets and improve the homogeneity (Lee et al., 2019).

Viscosity is a significant effect on the physicochemical properties and type (high viscosity: W/O or low viscosity: O/W) of nanoemulsion (Baboota et al., 2007). The droplet diameter affects the emulsion viscosity. The viscosity of nanoemulsion was found as 0.887 cP. A similar value (0.88 cP) was also reported for lemon nanoemulsion oil (Yazgan et al., 2019). Yazgan (2020) determined 1.51 cP for sage nanoemulsion (Yazgan, 2020), while in previous studies, the viscosity of essential oil was reported around 5 cP (Rao & McClements, 2012).

Also, Nepeta essential oil nanoemulsion showed acceptable stability during 2 months of storage at room temperature. The results are in agreement with those reported by previous authors (Guerra‐Rosas et al., 2016; Lee et al., 2019; Özogul et al., 2022; Yazgan, 2020; Zhang et al., 2017). Surfactants are added to the nanoemulsion system aiming at stability (Motta Felício et al., 2021). Furthermore, a high stability can be due to the small droplet size of Nepeta nanoemulsion (Guerra‐Rosas et al., 2016).

### 3.3 Free radical scavenging activity

**FIGURE 3:**
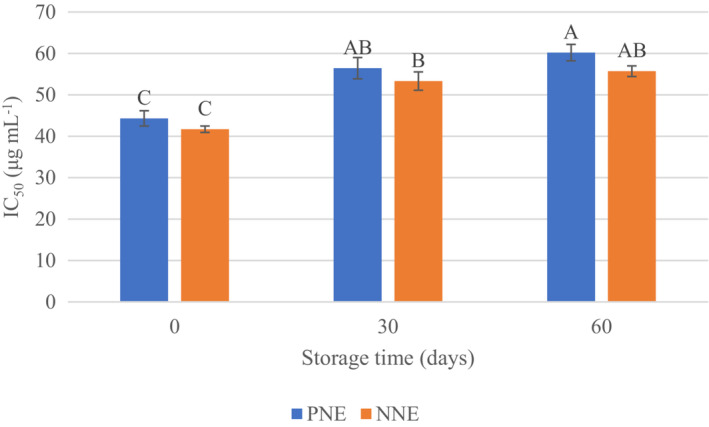
DPPH scavenging activity (expressed as IC_50_ (50% inhibitory concentration)) of the essential oil and nanoemulsion of *Nepeta pogonosperma*. Columns labeled with different letters are significantly different, *p* < .05 (*n* = 3). NNE, nanoemulsion of nepeta essential oil; PNE, pure Nepeta essential oil

The free radical scavenging properties of both pure *Nepeta pogonosperma* essential oil (PNE) and its nanoemulsified (NNE) were measured by the 2,2‐diphenyl‐1‐picrylhydrazyl (DPPH) assay based on IC_50_ values (Figure 3). Lower IC_50_ values present higher antiradical potential. The *Nepeta pogonosperma* essential oil, in pure and emulsified form, shows a relatively suitable antiradical potential during storage time (41.7–60.21 μg ml^−1^). The IC_50_ value of the *N. flavida* essential oil was also reported as around 43 μg ml^−1^ (Süntar et al., 2018). which is very close to the current results. Salehi et al. (2007) found that *N. ispahanica* extract exhibited relatively good radical scavenging activity with an IC_50_ value of 37 μg ml^−1^ (Salehi et al., 2007). However, Shakeri et al. (2016) indicated a poor antioxidant effect for *N. sintenisii* essential oil (IC_50_: 7.16 mg ml^−1^) (Shakeri et al., 2016). Moreover, lower antiradical potential (higher IC_50_) was reported for Nepeta extracts. For example, the IC_50_ value for *Nepeta cataria* methanol extract was 171.98 μg ml^−1^ (Afshar Mehrabi et al., 2021). Phenolic compounds are known as the main antioxidant and antiradical agents due to their hydrogen atom donation ability (Shahbazi, 2019). Various authors (Salehi et al., 2007; Sharifi & Khoshnoudi‐Nia, 2022; Sharifi‐Rad et al., 2020) proved a positive relationship between antioxidant activity and total phenolic concertation. Previous studies reported that 1,8‐cineole and nepetalactone were the main chemical composition of Nepeta essential oil with antiradical and antioxidant properties (Süntar et al., 2018; Tepe et al., 2007).

The pure essential oil showed higher IC_50_ value than nanoemulsion (53.66 vs. 49.90; *p* < .05). During storage, the antiradical performance of pure and emulsified essential oil was decreased (*p* < .05). However, emulsification can control the decreasing trend (*p* < .05). Investigation of various nanoformulations indicates that emulsification increased the antiradical potential of essential oils. In this regard, Seibert et al. (2019) showed that the nanoemulsion of *Cymbopogon densiflorus* essential oil has a more antiradical activity than its pure essential oil (*IC*_50_ values of 14.689 and 3.692 mg ml^−1^), respectively (Seibert et al., 2019). The IC_50_ value for the DPPH scavenging activity of pure and emulsified *Ocimum basilicum* essential oil was 13.21 and 10.47 μg ml^−1^ (Sundararajan et al., 2018). Moreover, the *IC*_50_ value of *Vitex negundo* essential oil and its nanoemulsion was 28.87 and 23.26 μg ml^−1^ (Balasubramani et al., 2017). It was demonstrated that nanostructures enhance the activity of biocompounds. Therefore, the nanoemulsified essential oil was able to reproduce bioactivity compared with pure essential oil even in lower concentrations (Balasubramani et al., 2017; Seibert et al., 2019).

### 3.4 Antibacterial activity

The antibacterial property of Nepeta essential oil and its nanoemulsion against Gram‐positive bacteria (*E. faecalis* and *B. cereus*) and Gram‐negative bacteria (*M. catarrhalis* and *K. pneumonia*) during 60 days of storage are given in Table 2. At the first time of storage, *Enterococcus faecalis* was the most susceptible bacteria to Nepeta essential oil and its nanoemulsion with 25.67 and 17.40 mm inhibition zone, respectively, while the lowest inhibition zone was recorded for *Klebsiella pneumoniae* (8.55 and 6.60 mm for PNE and NNE, respectively). However, Sage essential oil was more effective against *Klebsiella pneumoniae* with *an* inhibition zone value of 17.25 mm (Yazgan, 2020).

**TABLE 2:** The antibacterial property of *Nepeta pogonosperma* essential oil and its nanoemulsion against Gram‐positive bacteria (*E. faecalis* and *B. cereus*) and Gram‐negative bacteria (*M. catarrhalis* and *K. pneumonia*) during 60 days storage

Food‐borne pathogens	Inhabitation zone (mm)			
		1 day	30 days	60 days
*Enterococcus faecalis*	PNE	25.67 ± 1.51^A^	14.71 ± 0.94^C^	9.86 ± 1.06 ^E^
	NNE	17.40 ± 1.08^B^	14.68 ± 0.66^C^	12.33 ± 0.74^D^
*Bacillus cereus*	PNE	15.93 ± 1.42^A^	9.68 ± 0.88^C^	7.58 ± 0.66^D^
	NNE	12.30 ± 0.97^B^	9.43 ± 0.59^C^	8.80 ± 0.40^CD^
*Klebsiella pneumoniae*	PNE	8.55 ± 0.47^A^	6.30 ± 0.41^BC^	5.77 ± 0.47^C^
	NNE	6.60 ± 0.26^B^	6.47 ± 0.32^B^	6.23 ± 0.39^BC^
*Moraxella catarrhalis*	PNE	10.07 ± 0.50^A^	6.66 ± 0.43^CD^	5.31 ± 0.62 ^E^
	NNE	8.23 ± 0.33^B^	7.36 ± 0.41^C^	6.53 ± 0.22^D^

*Note*: For each bacteria strain, different letters show a significant difference *p* < .05 (*n* = 3).

Abbreviations: NNE, nanoemulsion of Nepeta essential oil; PNE, pure Nepeta nanoemulsion.

Low‐molecular‐weight compounds such as phenols, terpenes, terpenoids and other aromatic and aliphatic compounds can interact with the lipids of the bacterial cell membrane, increase the cell membrane permeability, and leakage the ions and cytoplasmic content, which affects the pH homeostasis system and leads to denaturant the enzyme, changes in cell morphology, and eventually death of the bacteria (Di Pasqua et al., 2007; Seow et al., 2014; Youseftabar‐Miri et al., 2021). It was proved that the antimicrobial properties of essential oils are related to their chemical composition, type of microbial strains, and interaction of essential oil and membrane of the microbial cell (Donsì & Ferrari, 2016). Sonboli et al. (2017) showed that *Nepeta hormozganica* had a suitable antibacterial against various *S*. epidermidis, *S. aureus*, and *E. coli* with an inhibition zone diameter of 24, 23, and 17 mm, respectively. The suitable antibacterial activity of the *N. menthoides* essential oil, besides 1,8‐cineole and 4aα‐7α‐7aα‐nepetalactone, was reported for *Bacillus cereus* (27, 26, and 25 mm, respectively); *Enterococcus faecalis* (16, 10, and 12 mm); and *Klebsiella pneumoniae* (16, 8, and 13 mm) by the previous author (Sonboli et al., 2009). In this regard, Sonboli et al. (2017) suggested that 1,8‐cineole and nepetalactone might be involved in the antimicrobial response of *Nepeta hormozganica* essential oil (Sonboli et al., 2017).

As seen in Table 2, on the first day of storage, the antibacterial effect of PNE (inhibition zone = 8.55–25.67 mm) was more than NNE (inhibition zone = 6.60–17.40 mm). Moghimi et al. (2017) reported that the antimicrobial potential of *Salvia officinalis* essential oil against *Haemophilus influenza* was higher than that recorded for the nanoemulsified one. However, for *Streptococcus pneumonia* and *Moraxella catarrhalis*, the nanoemulsion was much more effective than the pure essential oil (Moghimi et al., 2017). In addition, the superiority of the antibacterial capability of nanoemulsion as compared to essential oils has already been reported (Anwer et al., 2014; Maté et al., 2016; Yazgan et al., 2019). For example, Yazgan et al. (2019) reported that *E. faecalis* was more sensitive to lemon nanoemulsion (24.25 mm) than lemon essential oil (19.00 mm) (Yazgan et al., 2019). It can be related to the difference in bioactive compounds of essential oil, formulation, and particle size of emulsion as well as the type of bacterial strain (Donsì & Ferrari, 2016). Over time, nanoemulsion became more successful in maintaining its antibacterial effect (Table 2). This effect could be related to the increased surface area per mass of hydrophobic compounds formulated in nanoemulsions. These small oily droplets penetrate faster in the microbial membranes and improve the ability of bioactive compounds to interact with the microorganisms. It reduces the concentration of the essential oil needed to create a certain antibacterial effect (Donsì & Ferrari, 2016). Moreover, the restructuring of the bioactive materials within the nanoemulsion droplets and a higher local concentration of them could be a reason for the increased activity of nanoemulsion as compared to its essential oil (Garzoli et al., 2020).

### 3.5 Determination of minimum inhibition/bactericidal concentration (MIC and MBC)

Results of the MIC and MBC of the pure and emulsified nepeta essential oil on the food‐related pathogen microorganisms are summarized in Table 3. Both PNE and NNE were active against *E. faecalis* and *B. cereus* (Gram‐positive) while exhibiting a low inhibition effect on Gram‐negative *K. pneumoniae* and *M. cataharis*. The antibacterial activity of hydrocarbon and oxygenated monoterpenes (such as nepetalactone, 1,8‐cineole, linalool, α‐terpineol, and terpine‐4‐ol) was proved by previous studies (Badawy et al., 2019). The antibacterial ability of essential oil can be attributed to the ‐OH groups of bioactive compounds located at the *meta* and *ortho* positions. These OH‐ groups can interact with the cytoplasmic membrane of bacterial cells which leads to cell destruction and death (Shahbazi, 2019). *E. faecalis* was the most sensitive bacteria (MIC: 12.5 mg ml^−1^ and MBC: 25 mg ml^−1^). However, both pure and emulsified Nepeta essential oil did not show a great bactericidal capability on the *K. pneumunie* and *M. cataharis* (Gram‐negative bacteria), and the MIC and MBC values were of >50 mg ml^−1^, which was in agreement with previous studies (Ashrafi et al., 2020; Gharenaghadeh et al., 2017; Yazgan, 2020). Sonboli et al. (2017) showed that *Nepeta hormozganica* essential oil exhibited a moderate‐to‐strong antibacterial activity against *S. epidermidis*, *S. aureus*, and *E. coli*, and the effectiveness of this essential oil against Gram‐positive bacteria (e.g., *Staphylococcus epidermidis*) was higher than Gram‐negative bacteria (e.g., *Escherichia coli*). Moreover, Gharenaghadeh et al. (2017) also reported that MIC of *Salvia multicaulis* essential oil was 6.25 and 50 μg ml^−1^ for *E. faecalis* and *K. pneumoniae*, respectively. Yazgan (2020) reported that the bactericidal effect of sage essential oil against *E. faecalis* was low. However, Nepeta has a suitable bactericidal effect on Gram positive. The presence of the lipopolysaccharide layer in the structure of Gram‐negative bacteria is a barrier against the penetration of hydrophilic compounds (Youseftabar‐Miri et al., 2021; Zheng et al., 2017).

**TABLE 3:** Minimum inhibition concentration (MIC: mg ml^−1^) and minimum bactericidal concentration (MBC: mg ml^−1^) against food‐related pathogens

Microorganisms	Storage time (days)						
		0		30		60	
		MIC	MBC	MIC	MBC	MIC	MBC
*Enterococcus faecalis*	PNE	12.5	25	25	25	25	50
	NNE	12.5	12.5	25	25	50	50
*Basilus cereus*	PNE	25	25	25	25	50	50
	NNE	12.5	25	25	25	50	50
*Klebsiella pneumoniae*	PNE	50	>50	>50	>50	>50	>50
	NNE	50	>50	>50	>50	>50	>50
*Moraxella cataharis*	PNE	>50	>50	>50	>50	>50	>50
	NNE	50	>50	>50	>50	>50	>50

Abbreviations: NNE, nanoemulsion of Nepeta essential oil; PNE, pure Nepeta nanoemulsion.

The comparison of the antibacterial potential of Nepeta essential oil and its nanoemulsion showed that there was no significant difference between those at the first time. Over time, although the effectiveness of both essential oil and nanoemulsion significantly reduced, the decreasing trend was more pronounced in essential oil as compared to the nanoemulsion (Table 3). Emulsification of essential oil at the nanoscale increased the bioactivity through activation of the cell absorption mechanism (Nirmal et al., 2018). In this regard, Sundararajan et al. (2018) reported that *Ocimum basilicum* essential oil nanoemulsion had a lower MIC value as compared to the pure essential oil (for *Enterococcus faecalis*, *Staphylococcus aureus*, *Salmonella paratyphi*, and *Klebsiella pneumoniae*) (Sundararajan et al., 2018). Moreover, sage essential oil nanoemulsion showed a good antimicrobial effect against *S. typhi* PTCC 1609 (MIC = 8.0 mg ml), *E. coli* ATCC 25922 (MIC = 2.0 μg ml^−1^), and *S. dysentery* PTCC 1188 (MIC = 4.0 μg ml^−1^). However, the MIC and MBC values of pure sage essential oil were in the range 8.0–32.0 μg ml^−1^ against the same bacteria. Garzoli et al. (2020) also proved that lavandin essential oil nanoemulsion was more active against *E. coli* (MIC and MBC: 0.37 v/v%) and *B. cereus* (MIC and MBC: 0.09 and 0.19 v/v%) as compared to the pure essential oil (MIC for *E. coli*: 1.87 v/v% and MIC for *B. cereus*: 0.94 v/v%) (Garzoli et al., 2020). Therefore, nanoemulsions can be considered an effective delivery system for Nepeta essential oil.

## 4 CONCLUSION

The results show that on the first day of storage, the *Nepeta pogonosperma* essential oil has slightly more antioxidant and antibacterial potential than its nanoemulsion. However, the nanoemulsion of Nepeta essential oil was more successful in maintaining its bioactivity over time. The outcomes confirmed that the conversion of *Nepeta pogonosperma* essential oil into nanoemulsion improved the bioactivity of the essential oil. Decreasing the mean size of essential oil droplets within the nanoemulsion increases the local concentration of bioactive compounds and antioxidant and antimicrobial potentials of the nanoemulsion as compared to pure essential oil. It seems that NNE has great potential as a natural preservative to enhance the shelf‐life of food products, especially, in food products that Gram positive are dominant microorganisms. Further studies are needed to determine the mechanism action of PNE and NNE against food‐borne pathogens and spoilage bacteria and confirm the preservative effects of Nepeta nanoemulsion on food products.

## ACKNOWLEDGEMENTS

The authors gratefully acknowledge the support of Islamic Azad University, Qazvin, Iran.

## CONFLICT OF INTEREST

The authors declare that they have no conflict of interest.

## ETHICS STATEMENT

This article does not contain any studies with human participants or animals performed by any of the authors.

## Data Availability

The datasets generated and/or analyzed during the current study are available from the corresponding author upon reasonable request.
